# How does age affect the relationship between weight and health utility during the middle years of childhood?

**DOI:** 10.1007/s11136-018-1790-y

**Published:** 2018-02-05

**Authors:** Katie Eminson, Alastair Canaway, Peymané Adab, Emma Lancashire, Miranda Pallan, Emma Frew

**Affiliations:** 1 UK Centre for Tobacco and Alcohol Studies, University of Nottingham, Nottingham, NG7 2QL UK; 2 Warwick Clinical Trials Unit, University of Warwick, Coventry, CV4 7AL UK; 3 Public Health, University of Birmingham, Public Health Building, Birmingham, B15 2TT UK; 4 Health Economics Unit, University of Birmingham, Public Health Building, Birmingham, B15 2TT UK

**Keywords:** Health-related quality of life, Child health, Obesity, CHU-9D, Utility, Child weight status

## Abstract

**Purpose:**

The limited literature examining weight status and preference-based health-related quality of life (HRQL) in young children is equivocal. This study aims to examine how the association between weight status and preference-based HRQL changes as children develop between the ages of 6 and 10 years old.

**Methods:**

The Child Health Utility 9D (CHU-9D) was used to determine preference-based HRQL. Height and weight data were also collected and used to calculate z-BMI adjusted for age and gender. 1467 children were recruited from 54 schools across the West Midlands. Data were collected at four time points over 5 years. Impact of weight on dimensions of HRQL was assessed via the distribution of responses to CHU-9D dimensions by weight status. Multi-level regression analysis controlling for ethnicity, deprivation and other relevant co-variates was conducted to examine the relationship between weight and HRQL.

**Results:**

There was no evidence to suggest that the weight status impacted upon the distribution of responses to CHU-9D dimensions. Correspondingly, the multi-level regression analysis found no statistically significant differences in CHU-9D scores between underweight, healthy weight, overweight and obese children.

**Conclusions:**

The evidence surrounding the link between preference-based HRQL and weight status in children is limited. This study found no association between weight status and HRQL as measured by the CHU-9D in children between the ages of 5 and 10 years in the UK. Given this, it is recommended that future studies aiming to prevent obesity in children in their middle years do not rely solely on preference-based measures for economic evaluation, and instead focus on capturing clinical or wellbeing outcomes.

**Electronic supplementary material:**

The online version of this article (10.1007/s11136-018-1790-y) contains supplementary material, which is available to authorized users.

## Background

Economic evaluation acts as an aid to facilitate decision-making by providing information on the relative costs and effects of competing interventions [1]. A common application is to measure costs from a health service/social care perspective and to measure intervention effects using a metric termed quality-adjusted life years (QALYs) [1]. The advantage of measuring all outcomes using QALYs is that it facilitates comparisons of cost-effectiveness across interventions in multiple disease and clinical contexts [2]. QALYs provide a measure that encompasses both quantity and quality of life and for the same level of resource use, preference will be given to interventions that yield the greatest number of QALYs. Unlike general quality of life measures, a special characteristic of QALYs is that the quality of life scores (utilities) used to calculate QALYs are preference based [3].

There is growing consensus that early intervention is key to the prevention of childhood obesity [4]. However, in a world of scarce resources and limited public health budgets, it is also important to demonstrate cost-effectiveness using commonly applied outcome measures such as QALYs. The QALY metric rests on the assumption that, in comparison with full health, being in a ‘disease state’ (obesity) yields a lower quality of life and thus any gains from intervening and avoiding that health state will be realised in QALY gains over time. Understanding the relationship between health utility and childhood obesity is therefore very important for economic evaluation and resultant decision-making.

Of the few studies that have explored this, Frew et al. [5] found no evidence of a negative relationship between health utility and weight status in children aged 5–6 years or in children aged 6–7 years [6]. Both studies used the CHU-9D. Likewise, Belfort et al. [7] found no negative relationships in children and adolescents aged 5–18 years using the Health Utility Index—Mark 3. In contrast, however, a recent Australian study using the CHU-9D [8] did find a significant negative relationship between weight status and health utility in children aged 10 years, and Boyle et al. (2010) [9] found a significant negative relationship in 11–15-year-old children using the EQ-5D and a non-preference-based instrument, the PedsQL. The evidence for understanding this relationship is therefore inconsistent. Furthermore, to date, studies have been cross-sectional in nature. No study has followed the same cohort of children over time to examine whether the relationship changes as children develop and mature through their ‘middle years’. The middle years (age 6–12) is an important time to focus on, as it is particularly apt for childhood obesity prevention [10].

This study aimed to assess health utility in children followed up from age 5 to 10 years to examine how weight affects health utility as a child develops, and to evaluate the implications of this for resource allocation decision-making.

## Methods

### Data collection methods

The data within this study were collected as part of the West Midlands ActiVe lifestyle and healthy Eating in School children (WAVES) study. The WAVES study was a cluster-randomised, controlled trial based in the West Midlands, UK, which assessed the clinical effectiveness and cost-effectiveness of an obesity prevention intervention programme targeting young children. The study was funded by the National Institute for Health Research (ISRCTN97000586) and took place between 2010 and 2015 having obtained full ethics approval (NHS REC No. 10/H1202/69). The WAVES study was conducted in 54 primary schools spread across the West Midlands region with randomisation occurring at the school level. Data were collected at both the cluster (school) and within-cluster (individual pupils and their parents) levels [11].

A weighted random sampling process was used to select 54 schools from among 980 that were eligible. Sampling took account of the ethnic mix of pupils and was weighted to increase the likelihood of selecting schools that had a higher minority ethnic population with a 3:1 ratio to ensure that there would be sufficient power to detect differences across ethnicities [11]. All year 1 students at participating schools were eligible to take part, and an invitation letter was sent to all parents/carers of these children. Anthropometric (including height and weight) and health-utility data were collected from children with parental consent for study participation. Measurements were undertaken in school by trained researchers using standardised protocols. BMI values were calculated using the standard BMI calculation (weight in kg/height in m^2^). The weight-status groups were then formulated according to the UK 1990 BMI cut-offs [12] as recommended by NICE, UK [13]. Baseline (BL) data were collected when the children were in school year 1, aged 5–6 years. The intervention then ran for 1 year, and follow-up data were collected at 3 (F1), 18 (F2) and 30 (F3) months post intervention, with children aged 7–8, 8–9 and 9–10 years, respectively. Only half the schools were followed up at the final (30-month) follow-up point, as follow-up two was the primary end point of the trial [11].

The primary health-utility measure used was the Child Health Utility-9D (CHU-9D) instrument, designed for use within economic evaluations of interventions targeting paediatric populations [14]. The instrument was interviewer administered on a one-to-one basis. The CHU-9D consists of nine dimensions: worried; sad; annoyed; tired; pain; sleep; daily routine; schoolwork/homework and being able to join in activities, each of which has five severity levels. Each of the possible 1,953,125 health states described by the instrument can then be assigned a unique utility value ranging from 0.33 (worst health state) to 1 (best health state) using an algorithm that reflects the preference weight attached to each dimension [15]. These dimensions were originally identified through in-depth qualitative interviews with young people who had a variety of chronic and acute health problems to explore how their health affects their lives [14].

There are many potential methodological issues with measuring preference-based HRQL in children that has led to the validity of instruments being explored. Canaway and Frew [16] examined the performance of two GPBMs (the CHU-9D and the EQ-5D-Y [17]) in a pilot study for the WAVES trial, and found that although children aged 6–7 years could feasibly complete the instruments when administered by an interviewer, the reliability of the instruments was still uncertain. This pilot study concluded that the CHU-9D was the superior instrument, and it was recommended that the EQ-5D-Y should not be used to calculate utility values, until appropriate tariff values were available [16]. Frew et al. [5] contributed further evidence to the construct validity of the CHU-9D finding that HRQOL was associated with deprivation, and therefore the instrument was discriminating between these groups of children with known differences. Results from the same study also showed that the mean CHU9D values were significantly higher for children who reported individual PedsQL scores greater than the sample median PedsQL score [5]. Furthermore, Stevens [15] carried out a feasibility study and concluded that the CHU-9D could be used in the economic evaluation of paediatric interventions. For these reasons, the CHU9D was the instrument of choice for the WAVES study.

### Statistical analyses

Three analyses were undertaken to explore the relationship between weight status and health-utility values over the study period.

First, to explore the relationship between health utility and weight status, descriptive statistics are reported. The paper reports the mean health-utility values (and SD) by weight status at baseline and follow-up points one, two and three as the children age.

Second, to examine the impact of weight status on the dimensions of health, the distributions of responses to each dimension of the CHU-9D were examined by weight status. For this analysis, weight status was divided into two groups—‘healthy/underweight’ and ‘overweight/obese’. The Chi-squared test was used to examine whether there were any differences between the patterns of responses in the two groups.

Finally, to model the relationship between health utility and weight status, a multi-level regression model, with random effects at the school level, was developed. The model controlled for potential confounders including gender, ethnicity, and deprivation, where deprivation was based on the IMD scores derived from the home postcode [11]. These co-variates were selected as prior research suggests they are associated with health-related quality of life [5, 18, 19]. It was hypothesised that higher utility scores would be observed in children of a healthy weight compared to those who are overweight or obese and that this relationship would become stronger as children age. There is some evidence that physical activity has an impact on HRQL in children, i.e. overweight children who are more active may have a greater HRQL than their peers who have a healthy weight but are less active [20]. We checked this by including a variable for exercise which used accelometery data. We ran the model with and without data for F3 to check whether missing data were an issue. The analysis was undertaken using Stata 13 [21].

## Results

The baseline sample was 1397 children (mean age = 6 years) of which 51% were male. The trial design necessitated that only half the cohort was followed up at the final time point, and there was also attrition throughout the study. Therefore, the participants measured at follow-up time points were *n*(F1) = 1252, *n*(F2) = 1141 and *n*(F3) = 489 with corresponding sample mean ages of, 8, 9 and 10 years, respectively. The study population had a diverse ethnic and socioeconomic mixes with 55% being non-white and 55% from the most-deprived quintile. Participant characteristics are shown in Table 1.

**Table 1:** Characteristics of trial participants

Characteristics	Baseline	Follow-up 1	Follow-up 2	Follow-up 3
Age: Mean (SD) (*n*=)	6.2 (0.307) (*n* = 1397)	7.6 (0.298) (*n* = 1252)	8.9 (0.302) (*n* = 1141)	9.6 (0.306) (*n* = 489)
Weight status: *n* (%)				
Underweight	40 (2.87)	37 (2.96)	32 (2.81)	8 (1.67)
Healthy weight	1055 (75.79)	879 (70.43)	743 (65.29)	298 (62.21)
Overweight	125 (8.98)	140 (11.22)	143 (12.57)	68 (14.20)
Obese	172 (12.36)	192 (15.38)	220 (19.33)	105 (21.92)
CHU-9D: Mean (SD) (*n*=)	0.826 (0.139) (*n* = 1350)	0.863 (0.107) (*n* = 1215)	0.896 (0.091) (*n* = 1128)	0.905 (0.088) (*n* = 485)
Underweight	0.855 (0.131) (*n* = 38)	0.825 (0.13) (*n* = 37)	0.898 (0.1) (*n* = 32)	0.908 (0.07) (*n* = 8)
Healthy weight	0.826 (0.14) (*n* = 1022)	0.863 (0.11) (*n* = 852)	0.897 (0.09) (*n* = 729)	0.904 (0.09) (*n* = 295)
Overweight	0.805 (0.143) (*n* = 118)	0.866 (0.1) (*n* = 138)	0.899 (0.09) (*n* = 140)	0.911 (0.09) (*n* = 68)
Obese	0.829 (0.128) (*n* = 167)	0.865 (0.11) (*n* = 182)	0.89 (0.1) (*n* = 217)	0.908 (0.09) (*n* = 104)
Gender: *n* (%) (*n* = 1467)				
Male	749 (51.06)			
Female	718 (48.94)			
Ethnicity: *n* (%)				
White	658 (45.38)			
South Asian	443 (30.53)			
Black African Caribbean	115 (7.93)			
Other	235 (16.20)			
Deprivation quintile: *n* (%)				
Most deprived	803 (55.53)			
2	261 (18.05)			
3	164 (11.34)			
4	122 (8.44)			
Least deprived	96 (6.64)			

Table 2 shows the distribution of CHU-9D dimensions by weight status at the three follow-up points. Overall the majority of the population had no or very few problems to report across all CHU-9D dimensions regardless of their weight status. The only dimension for which a statistically significant difference in the distribution of responses between the underweight/healthy weight and the overweight/obese groups was indicated was for ability to join in activities (‘Activities’), and this was only evident at the final follow-up (mean age of 10 years).

**Table 2:** Distribution of response to CHU-9D dimensions by weight status

CHU-9D dimensions	Level	Follow-up 1			Follow-up 2			Follow-up 3		
		Underweight/healthy (*n* = 895) *n* (%)	Overweight/obese (*n* = 325) *n* (%)	Chi square *p* value	Underweight/healthy (*n* = 769) *n* (%)	Overweight/obese (*n* = 360) *n* (%)	Chi square *p* value	Underweight/healthy (*n* = 306) *n* (%)	Overweight/obese (*n* = 172) *n* (%)	Chi square *p* value
Worried	No	647 (72.29)	220 (67.69)	0.232	617 (80.23)	294 (81.67)	0.16	271 (88.56)	147 (85.47)	0.455
	A little bit	128 (14.30)	59 (18.15)		94 (12.22)	32 (8.89)		23 (7.52)	18 (10.47)	
	A bit	53 (5.92)	24 (7.38)		40 (5.20)	18 (5.00)		8 (2.61)	4 (2.33)	
	Quite	32 (3.58)	14 (4.31)		16 (2.08)	13 (3.61)		2 (0.65)	3 (1.74)	
	Very	35 (3.91)	8 (2.46)		2 (0.26)	3 (0.83)		2 (0.65)	0 (0.00)	
Sad	No	674 (75.31)	241 (74.15)	0.294	654 (85.16)	291 (80.83)	0.443	267 (87.54)	152 (87.86)	0.654
	A little bit	124 (13.85)	49 (15.08)		74 (9.64)	43 (11.94)		30 (9.84)	14 (8.09)	
	A bit	39 (4.36)	9 (2.77)		23 (2.99)	14 (3.89)		4 (1.31)	4 (2.31)	
	Quite	28 (3.13)	17 (5.23)		11 (1.43)	7 (1.94)		3 (0.98)	1 (0.58)	
	Very	30 (3.35)	9 (2.77)		6 (0.78)	5 (1.39)		1 (0.33)	2 (1.16)	
Pain	No	594 (66.37)	211 (64.92)	0.346	523 (68.01)	256 (71.11)	0.124	194 (63.40)	115 (66.47)	0.512
	A little bit	182 (20.34)	79 (24.31)		164 (21.33)	57 (15.83)		74 (24.18)	42 (24.28)	
	A bit	55 (6.15)	17 (5.23)		51 (6.63)	32 (8.89)		20 (6.54)	12 (6.94)	
	Quite	32 (3.58)	12 (3.69)		22 (2.86)	8 (2.22)		10 (3.27)	2 (1.16)	
	Very	32 (3.58)	6 (1.85)		9 (1.17)	7 (1.94)		8 (2.61)	2 (1.16)	
Tired	No	412 (46.03)	147 (45.23)	0.859	382 (49.67)	187 (51.94)	0.652	133 (43.46)	88 (50.87)	0.244
	A little bit	258 (28.83)	92 (28.31)		219 (28.48)	99 (27.50)		111 (36.27)	56 (32.37)	
	A bit	86 (9.61)	30 (9.23)		88 (11.44)	32 (8.89)		38 (12.42)	23 (13.29)	
	Quite	60 (6.70)	28 (8.62)		40 (5.20)	23 (6.39)		11 (3.59)	2 (1.16)	
	Very	79 (8.83)	28 (8.62)		40 (5.20)	19 (5.28)		13 (4.25)	4 (2.31)	
Annoyed	No	699 (78.10)	252 (77.78)	0.683	620 (80.62)	290 (80.56)	0.873	260 (84.97)	147 (84.97)	0.993
	A little bit	103 (11.51)	31 (9.57)		79 (10.27)	32 (8.89)		25 (8.17)	14 (8.09)	
	A bit	30 (3.35)	15 (4.63)		37 (4.81)	19 (5.28)		11 (3.59)	7 (4.05)	
	Quite	19 (2.12)	7 (2.16)		14 (1.82)	9 (2.50)		5 (1.63)	3 (1.73)	
	Very	44 (4.92)	19 (5.86)		19 (2.47)	10 (2.78)		5 (1.63)	2 (1.16)	
School/home work	No problems	508 (56.82)	188 (58.20)	0.825	513 (67.06)	239 (66.76)	0.689	221 (72.46)	124 (71.68)	0.578
	A few	254 (28.41)	89 (27.55)		185 (24.18)	88 (24.58)		64 (20.98)	39 (22.54)	
	Some	81 (9.06)	25 (7.74)		52 (6.80)	22 (6.15)		12 (3.93)	6 (3.47)	
	Many	27 (3.02)	9 (2.79)		6 (0.78)	6 (1.68)		2 (0.66)	3 (1.73)	
	Can’t do	24 (2.68)	12 (3.72)		9 (1.18)	3 (0.84)		6 (1.97)	1 (0.58)	
Sleep	No problems	475 (53.31)	172 (53.42)	0.174	482 (63.17)	227 (63.23)	0.396	191 (62.62)	115 (66.47)	0.647
	A few	185 (20.76)	71 (22.05)		173 (22.67)	73 (20.23)		74 (24.26)	40 (23.12)	
	Some	57 (6.40)	31 (9.63)		37 (4.85)	24 (6.69)		17 (5.57)	6 (3.47)	
	Many	48 (5.39)	13 (4.04)		30 (3.93)	10 (2.79)		12 (3.93)	4 (2.31)	
	Can’t do	126 (14.14)	35 (10.87)		41 (5.37)	25 (6.96)		11 (3.61)	8 (4.62)	
Daily routine	No problems	707 (79.08)	250 (77.40)	0.319	658 (85.90)	306 (85.00)	0.971	275 (89.89)	155 (89.60)	0.385
	A few	106 (11.86)	43 (13.31)		77 (10.05)	39 (10.83)		22 (7.19)	16 (9.25)	
	Some	40 (4.47)	19 (5.88)		22 (2.87)	11 (3.06)		6 (1.96)	0 (0.00)	
	Many	18 (2.01)	8 (2.48)		3 (0.39)	2 (0.56)		2 (0.65)	1 (0.58)	
	Can’t do	23 (2.57)	3 (0.93)		6 (0.78)	2 (0.56)		1 (0.33)	1 (0.58)	
Activities	All	582 (65.03)	228 (70.37)	0.419	542 (70.63)	230 (63.89)	0.181	236 (77.12)	125 (72.25)	0.037**
	Most	173 (19.33)	54 (16.67)		140 (18.28)	86 (23.89)		49 (16.01)	35 (20.23)	
	Some	65 (7.26)	23 (7.10)		43 (5.61)	25 (6.94)		8 (2.61)	10 (5.78)	
	A few	50 (5.59)	13 (4.01)		30 (3.92)	13 (3.61)		12 (3.92)	1 (0.58)	
	None	25 (2.79)	6 (1.85)		12 (1.57)	6 (1.67)		1 (0.33)	2 (1.16)	

**Significant at *p* = 0.05

The results of the multi-level linear regression to examine the impact of weight status on health utility whilst controlling for potential confounders are shown in Table 3. There was no statistically significant difference in health-utility values between those in different weight status groups indicating that for the age groups considered, there is no association between health utility and weight status. The coefficient for age, however, was statistically significant (0.024 95% CI 0.022–0.027), and furthermore, deprivation was also significant with children in the most-deprived groups having a lower health utility than those in the least-deprived group. When F3 data were omitted from the model, the results were near identical (see supplementary materials Table 1).

**Table 3:** Regression results from analyses investigating the impact of weight status on health utility

CHU-9D score	Coef.	*p* value	95% Conf. Interval	
			Lower CI	Upper CI
Age	0.024292	0.000**	0.021559	0.027024
Gender^a^				
Female	0.001701	0.634	− 0.00529	0.008692
Weight status^b^				
Healthy weight	0.000437	0.968	− 0.02068	0.021551
Overweight	− 0.00126	0.915	− 0.02438	0.021864
Obese	0.003166	0.782	− 0.01924	0.025576
Ethnicity^c^				
Asian	− 0.00174	0.739	− 0.01196	0.008478
African Caribbean	− 0.00843	0.272	− 0.02346	0.006601
Other	− 0.00159	0.782	− 0.01284	0.009661
Not known	0.002852	0.936	− 0.06716	0.072859
IMD quintile^d^				
2	0.010348	0.064	− 0.00062	0.021312
3	0.010426	0.108	− 0.0023	0.023146
4	0.001092	0.886	− 0.01386	0.016043
Least deprived	0.029459	0.001**	0.011988	0.04693
_cons	0.670001	0**	0.638859	0.701143

Reference categories: ^a^Male, ^b^Underweight, ^c^White, ^d^Most deprived

**Significant at *p* = 0.05

When we included the variable for exercise, the coefficient was very small and non-significant, implying that exercise itself does not have a significant impact on children’s HRQL (see supplementary materials Table 2).

## Discussion

With childhood obesity projected to rise, there have been attempts to address this growing public health concern through the promotion of healthy lifestyles and through obesity prevention and management schemes. This study contributes to the growing body of research in childhood obesity and the relationship between weight status and preference-based health utility over a period of time using the specially designed child utility instrument, the CHU-9D, within an ethnically and socioeconomically diverse population within the UK.

The main aim of this study was to investigate the longitudinal relationship between weight status and health utility in children during the middle-years phase. The results suggest there is no statistically significant relationship between weight status and health utility when measured using the CHU-9D during this time period. After adjusting for known potential confounders, for all ages, the relationship between health utility and weight status was negligible and not statistically significant. The study also found little evidence of differences in the distributions of response to the CHU-9D dimensions by weight status as the children aged. One dimension (activities) at the final follow-up was significantly different between the two groups. Given the consistent lack of evidence for differences between the two groups with regard to all other dimensions at all other time points; the smaller sample size at this time point resulting in low numbers in several cells; and the use of multiple testing, this outcome should be interpreted with caution. These results support the findings of three studies [5–7] where no link was found, however, differing from the findings of two other papers [8, 9] which suggest an association.

As well as the main outcome of the effect of obesity on reported health utility as children grow older, another interesting finding emerged. Health utility was lower for children who were in the most-deprived quintile. This result indicates that the CHU-9D is capable of discriminating between subgroups of children based on deprivation.

One of the study’s main strengths is its design. First, all height and weight measurements were undertaken by trained researchers using standardised equipment and operating procedures to ensure consistency in measurement. Similarly, the CHU-9D was interviewer administered to ensure that the children engaged with the task. Unlike previous studies of the effect of weight status on health utility, this study included a longitudinal element which allowed for an examination of how that relationship changed during the middle years.

The main weakness of the study surrounds the difficulty in deciphering whether there is truly no relationship between weight status and health utility as children grow older from 6 to 10 years, or whether the CHU-9D is simply not sensitive enough to identify one. The CHU-9D was originally designed for use with children of 7–11 year olds [22], and although Canaway and Frew [16] found the instrument to be acceptable and feasible for children aged 6–7 years, there are still concerns regarding the instrument’s reliability [16, 23] in younger children. There exists evidence on the relationship between weight status and HRQL in the non-preference HRQL literature. Ottova et al. [24] used data from the KIDSCREEN health interview survey [25] which included over 17,000 children and adolescents aged 8–18 years from 10 European countries. Results of this study support the hypothesis that overweight or obese children and adolescents have a lower HRQL than their healthy-weight counterparts. And, these results are consistent with other HRQL studies [26–29] that used non-preference-based instruments and found evidence that paediatric obesity impacts on self-esteem and quality of life.

The finding that HRQL increases systematically with children getting older is interesting, and even though the analysis included known potential confounders, it is possible that there were other unknown factors that were omitted.

This result has implications for the economic evaluation of childhood obesity interventions. One could argue that having no negative effect identified by preference-based measures is not necessarily a concern for economic evaluation as long-term modelling that extrapolates results into the future could account for future cost-savings and benefit from preventing obesity into adulthood. Whilst this might be true, the reality is that the clinical and public health evidence base is strongly in favour of preventing childhood obesity, and if the conventional application of measuring QALYs is pursued in individuals in the middle-years phase of childhood, then any gains from intervening are not going to be realised in QALY terms. As a result, conventional rules for judging whether an intervention is deemed cost-effective using a cost/QALY ratio may disadvantage interventions targeting childhood obesity.

## Electronic supplementary material

Below is the link to the electronic supplementary material.

Supplementary material 1 (DOCX 19 KB)
