# Supplementary material for: How does age affect the relationship between weight and health utility during the middle years of childhood?
Source: Qual Life Res. 2018 Feb 5;27(6):1455–62. doi: 10.1007/s11136-018-1790-y (PMC5951869; doi:10.1007/s11136-018-1790-y)
Supplement: Supplementary file 1 — Supplementary material 1 (DOCX 19 KB) [file 11136_2018_1790_MOESM1_ESM.docx]

# Supplementary Analyses

Supplementary Table 1: Including only data up to follow up 2

| **CHU-9D** | **Coef.** | **P>z** | **95% Conf.**  **Interval** | |
| --- | --- | --- | --- | --- |
|  |  |  | **lower CI** | **upper CI** |
| **Age** | 0.025367 | 0^**^ | 0.022112 | 0.028622 |
|  |  |  |  |  |
| **Gender^1^** |  |  |  |  |
| **Female** | 0.001664 | 0.668 | -0.00594 | 0.009268 |
|  |  |  |  |  |
| **Weight Status^2^** |  |  |  |  |
| **Normal** | 0.000026 | 0.998 | -0.02243 | 0.02248 |
| **Overweight** | -0.00357 | 0.777 | -0.02835 | 0.021207 |
| **Obese** | 0.001651 | 0.893 | -0.02231 | 0.025616 |
|  |  |  |  |  |
| **Ethnicity^3^** |  |  |  |  |
| **Asian** | -0.00269 | 0.632 | -0.01369 | 0.008315 |
| **African Caribbean** | -0.00983 | 0.234 | -0.02604 | 0.006374 |
| **Other** | -0.00072 | 0.909 | -0.013 | 0.011569 |
| **Not known** | 0.002632 | 0.943 | -0.06919 | 0.074453 |
|  |  |  |  |  |
| **IMD Quintile^4^** |  |  |  |  |
| **2** | 0.009935 | 0.102 | -0.00199 | 0.021857 |
| **3** | 0.014456 | 0.042^**^ | 0.00052 | 0.028393 |
| **4** | 0.000623 | 0.939 | -0.01546 | 0.016712 |
| **Least deprived** | 0.029626 | 0.002^**^ | 0.011059 | 0.048192 |
|  |  |  |  |  |
| **_cons** | 0.663135 | 0^**^ | 0.628549 | 0.697721 |

^Reference categories: 1 = Male, 2 = Underweight, 3 = White, 4 = Most deprived. **Significant at p=0.05^

Supplementary Table 2: Including exercise within the model

| **CHU-9D Score** | **Coef.** | **P>z** | **95% Conf.**  **Interval** | |
| --- | --- | --- | --- | --- |
|  |  |  | **Lower CI** | **Upper CI** |
| **Age** | 0.02541 | 0^**^ | 0.021676 | 0.0291446 |
|  |  |  |  |  |
| **Gender^1^** |  |  |  |  |
| **Female** | 0.007982 | 0.086 | -0.00112 | 0.0170894 |
|  |  |  |  |  |
| **Weight Status^2^** |  |  |  |  |
| **Normal** | -0.00168 | 0.895 | -0.02673 | 0.023358 |
| **Overweight** | 0.001971 | 0.89 | -0.02604 | 0.0299807 |
| **Obese** | -0.00112 | 0.935 | -0.02808 | 0.0258452 |
|  |  |  |  |  |
| **Ethnicity^3^** |  |  |  |  |
| **Asian** | -0.0026 | 0.682 | -0.01502 | 0.0098335 |
| **African Caribbean** | -0.00382 | 0.7 | -0.02325 | 0.0156049 |
| **Other** | 0.000868 | 0.901 | -0.01285 | 0.0145826 |
| **Not known** | -0.01471 | 0.769 | -0.11307 | 0.0836485 |
|  |  |  |  |  |
| **IMD Quintile^4^** |  |  |  |  |
| **2** | 0.011709 | 0.088 | -0.00174 | 0.0251615 |
| **3** | 0.014098 | 0.075 | -0.00142 | 0.0296146 |
| **4** | 0.002841 | 0.754 | -0.01496 | 0.0206409 |
| **Least deprived** | 0.038106 | 0^**^ | 0.017166 | 0.0590466 |
|  |  |  |  |  |
| **Exercise** | 0.000115 | 0.226 | -7.1E-05 | 0.0003018 |
| **_cons** | 0.652307 | 0^**^ | 0.60844 | 0.6961735 |

^Reference categories: 1 = Male, 2 = Underweight, 3 = White, 4 = Most deprived. **Significant at p=0.05^
